# Animal-assisted interventions for depression in older adults: an umbrella review with meta-analysis and trial sequential analysis

**DOI:** 10.1007/s40520-026-03454-9

**Published:** 2026-07-04

**Authors:** Marina De Rui, Alessandro Perencin, Mario Virgilio Papa, Lisa Doria, Adele Ravelli, Chiara Ceolin, Andrea Ungar, Giuseppe Sergi, Maria Devita

**Affiliations:** 1 Geriatrics Division, Department of Medicine (DIMED), University of Padua, Padua, Italy; 2 Department of Neurobiology, Care Sciences and Society, Aging Research Center, Karolinska Institutet and Stockholm University, Stockholm, Sweden; 3 Department of General Psychology, University of Padua, Padova, Italy; 4 Department of Critical Care Medicine and Surgery, Unit of Gerontology and Geriatrics - Hypertension Centre, University of Florence and Azienda Ospedaliera Careggi, Florence, Italy

**Keywords:** Animal-assisted therapy, Pet therapy, Depression, Older adults, Elderly, Umbrella review, Trial sequential analysis

## Abstract

**Background:**

Depression affects up to 30% of older adults and is associated with increased morbidity and mortality. Animal-assisted interventions (AAIs) - usually known as “pet therapy” - have emerged as promising complementary approaches, yet evidence synthesis remains fragmented.

**Objective:**

To comprehensively evaluate the effectiveness of AAI in reducing depressive symptoms among older adults through an umbrella review of systematic reviews and meta-analyses, incorporating trial sequential analysis.

**Methods:**

We systematically searched PubMed, Embase, and Cochrane Library through September 2025 for systematic reviews and meta-analyses examining AAIs for depression in older adults. Quality assessment was performed using AMSTAR-2. We calculated the Corrected Covered Area to assess overlap between meta-analyses, re-analyzed primary study data to avoid double-counting, and conducted trial sequential analysis to evaluate evidence sufficiency.

**Results:**

Five systematic reviews encompassing 27 unique primary studies with 2,156 participants were included. The pooled random-effects meta-analysis demonstrated a significant reduction in depressive symptoms (SMD = 0.73; 95% CI: 0.57–0.89; *p* < 0.001) with moderate heterogeneity (I² = 43.4%). Trial sequential analysis revealed that the cumulative Z-curve crossed monitoring boundaries at 75% of the required information size, confirming evidence sufficiency. The Corrected Covered Area was 20.4%, indicating moderate overlap between meta-analyses.

**Conclusions:**

AAIs are associated with clinically meaningful reductions in depressive symptoms among older adults. Trial sequential analysis confirms that sufficient evidence exists to support this conclusion. AAIs represent a valuable, low-risk, patient-centered complement to conventional depression treatments in geriatric care.

**PROSPERO registration:**

[CRD420251250448]

**Supplementary Information:**

The online version contains supplementary material available at https://doi.org/10.1007/s40520-026-03454-9.

## Introduction

Late-life depression is one of the most prevalent and disabling conditions in older adults, affecting up to 30% of individuals aged 65 years and older [1, 2]. It is strongly associated with frailty, cognitive impairment, hospitalization, and mortality [1, 3–5], exerting a profound impact on functional status and quality of life. Despite its clinical relevance, late-life depression remains frequently underdiagnosed and inadequately treated, partly because its symptoms often overlap with age-related changes, multimorbidity, and the tendency of older adults to express emotional distress through somatic complaints. These challenges underscore the need for complementary, multidimensional strategies that extend beyond pharmacotherapy and integrate psychosocial and behavioral interventions [6, 7]. 

Among these approaches, animal-assisted interventions (AAIs)—commonly referred to as “pet therapy”—have gained increasing attention as non-pharmacological strategies in geriatric care. AAIs are used in a wide range of settings, including nursing homes, rehabilitation units, and community programs, where structured interactions with trained animals aim to reduce psychological distress, alleviate loneliness, and foster emotional engagement. AAIs integrate structured interactions with trained animals to address multiple dimensions of late-life depression: reducing psychological distress, alleviating loneliness, enhancing social participation, and providing gentle physical and cognitive stimulation [8–15]. 

Over the past two decades, numerous studies have investigated the impact of AAIs on depressive symptoms among older adults [10, 16–24]. Several systematic reviews and meta-analyses have synthesized these findings, but conclusions remain inconsistent due to methodological heterogeneity, differences in intervention protocols and outcome measures, and variability in populations and settings [8]. 

Given the growing interest in non-pharmacological strategies for late-life depression—particularly in the context of multimorbidity, polypharmacy, and limited treatment adherence—a clearer and more integrated understanding of the effectiveness of AAIs is needed. This umbrella review therefore aims to provide a comprehensive synthesis of the highest-level evidence on the role of pet therapy in reducing depressive symptoms in older adults. By accounting for study overlap and applying trial sequential analysis, we seek to clarify the magnitude and consistency of reported effects and to inform clinical practice and future research on the therapeutic potential of AAIs within holistic geriatric care models.

## Materials and methods

The Preferred Reporting Items for Systematic Reviews and Meta-analyses (PRISMA) 2020 guidelines were used to guide this umbrella review. The full protocol was prospectively registered in PROSPERO (registration number: CRD420251250448).

### Data sources and searches

A comprehensive search was conducted in Embase, PubMed, and the Cochrane Library from inception to September 2025 for systematic reviews and meta-analyses. The search strategy combined key terms related to animal-assisted interventions (e.g., animal-assisted therapy, pet therapy, therapy dog), depression (e.g., depression, depressive symptoms, depressive disorder), and older adults (e.g., elderly, older adults, geriatric populations). No language restrictions were applied. Reference lists of eligible articles were hand-searched for additional relevant studies.

### Study selection

For the purpose of this umbrella review, we included: (i) systematic reviews and meta-analyses that assessed longitudinal observational studies (prospective or retrospective) evaluating the impact of animal-assisted interventions on depressive symptoms in older adults; and (ii) systematic reviews and meta-analyses investigating interventional studies comparing different types of animal-assisted programs or their effectiveness versus standard care or control conditions in older adults.

We excluded systematic reviews based solely on cross-sectional studies, narrative reviews lacking a structured literature search, conference abstracts, meta-analyses including fewer than two studies per outcome, letters to the editor, and editorials. In addition, studies not published in English or not formally published in peer-reviewed journals were not considered. When multiple systematic reviews addressed the same clinical question using similar study designs and populations, we selected the one including the largest number of eligible primary studies to avoid redundancy while ensuring comprehensive coverage.

### Data extraction

Two reviewers (AP and MVP) independently screened titles and abstracts for eligibility. In cases of disagreement, a third senior reviewer (CC) was consulted to reach consensus. Full texts of potentially eligible articles were then retrieved and independently assessed for inclusion by the same two reviewers (AP and MVP). Discrepancies were again resolved through discussion with the senior reviewer (CC).

The following information was extracted from each eligible study using a standardized data extraction form: first author and year of publication, country of origin, type of review (systematic review or meta-analysis), number and design of included primary studies (randomized controlled trials, quasi-experimental studies, pre-post studies), total number of participants, participant characteristics (age range, gender distribution, care setting), type of animal-assisted intervention (animal species, intervention duration, frequency, and format), control or comparison conditions, depression assessment tools used, main findings (effect sizes with 95% confidence intervals), measures of heterogeneity (I² statistics, τ² values), and reported limitations.

We also extracted data required for the Assessment of Multiple Systematic Reviews (AMSTAR-2) tool quality assessment.

To quantify the degree of overlap between meta-analyses, we calculated the Corrected Covered Area (CCA) for each outcome following established methodological guidelines. The CCA was computed as: CCA = (N - r) / (r × c - r), where N represents the total number of primary study entries across all meta-analyses, r is the number of unique primary studies, and c is the number of meta-analyses. Outcomes with a high degree of overlap (CCA ≥ 15%) were re-analyzed using unique primary study data to avoid double-counting and overestimation of precision.

### Quality assessment

Two reviewers (AP and MVP) independently assessed the methodological quality of the included meta-analyses using the AMSTAR-2 tool, which evaluates systematic reviews across sixteen predefined domains. These domains assess protocol registration, adequacy of literature search, justification for study design inclusion, duplicate study selection and data extraction, list of excluded studies with justification, adequate description of included studies, risk of bias assessment, appropriateness of meta-analytic methods, consideration of risk of bias in interpreting results, assessment of publication bias, reporting of conflicts of interest, and overall transparency. Based on performance across critical and non-critical domains, overall quality was rated as high, moderate, low, or critically low. Disagreements were resolved through discussion with a senior reviewer (CC). Detailed item-level ratings for each review are reported in Supplementary Table S1.

### Data synthesis and analysis

Where quantitative data were available, pooled effect estimates (standardized mean differences with corresponding 95% confidence intervals) were not only extracted from the original meta-analyses but also recalculated by retrieving data from the individual primary studies included in those meta-analyses. This re-analysis allowed a more consistent and comparable synthesis across reviews and minimized potential overlap-related biases.

Effect sizes were interpreted according to Cohen’s conventions: SMD values of 0.2, 0.5, and 0.8 representing small, moderate, and large effects, respectively. Measures of statistical heterogeneity (I² statistics and p-values from Cochran’s Q test) were reported to assess consistency across studies. The I² statistic was interpreted according to the Cochrane Handbook recommendations proposed by Higgins et al., where values of 0–40% may not be important, 30–60% may represent moderate heterogeneity, 50–90% substantial heterogeneity, and 75–100% considerable heterogeneity.

These categories are intended as rough guides and should be interpreted in conjunction with the magnitude and direction of effects and the strength of evidence for heterogeneity.When multiple meta-analyses addressed the same outcome, findings were compared in terms of direction, magnitude of effect, and methodological quality. The 95% prediction interval was calculated to estimate the range of true effects that could be expected in future studies, providing insight into the uncertainty and generalizability of the findings.

The meta-analysis was performed using Review Manager (RevMan Version 5.4, The Cochrane Collaboration, 2020) with a random-effects model based on the DerSimonian-Laird method, which accounts for both within-study and between-study variability. Sensitivity analyses were conducted by sequentially removing each study to assess the robustness of the pooled estimate. Influential studies were identified using standardized residuals, DFFITS values, Cook’s distance, and changes in heterogeneity (τ²) upon study removal.

### Grading the evidence

When the p-value for the random-effects model was < 0.05, we evaluated the quality of evidence from randomized controlled trials using the GRADE (Grading of Recommendations, Assessment, Development and Evaluation) framework. Evidence quality was assessed across five domains: risk of bias, inconsistency, indirectness, imprecision, and publication bias. Evidence could be rated down if serious concerns were identified in any domain. Additionally, we considered several factors as potential indicators of bias in the available evidence, including whether the 95% prediction interval excluded the null value, the presence of substantial heterogeneity (I² > 50%), small-study effects assessed through visual inspection of funnel plots and Egger’s regression test (*p* < 0.10), and evidence of excess significance bias (*p* < 0.10). A detailed GRADE evidence profile, including judgments across risk of bias, inconsistency, indirectness, imprecision, and publication bias, is reported in Supplementary Table S2.

### Trial sequential analysis

To determine whether the cumulative evidence was sufficient to draw reliable conclusions and to control for type I error inflation due to repeated significance testing in meta-analyses, we conducted Trial Sequential Analysis (TSA) using TSA software version 0.9.5.10 Beta (Copenhagen Trial Unit, Denmark).

The required information size was calculated assuming a minimal clinically relevant difference of SMD = 0.5 (representing a moderate effect size), with alpha set at 0.05 (two-sided), power (1-beta) at 80%, and variance estimated from the included studies. Trial sequential monitoring boundaries were constructed to account for the risk of false-positive findings from accumulating evidence. The cumulative Z-curve was plotted against these boundaries to assess whether sufficient evidence had been accrued to detect or refute the anticipated intervention effect.

If the Z-curve crossed the trial sequential monitoring boundary before reaching the required information size, this would indicate that sufficient evidence existed to conclude efficacy without additional studies. Conversely, if the Z-curve entered the futility area, this would suggest that the intervention effect was smaller than the minimal relevant difference, and further research might be unnecessary.

## Results

### Study selection and characteristics

**Fig. 1:**
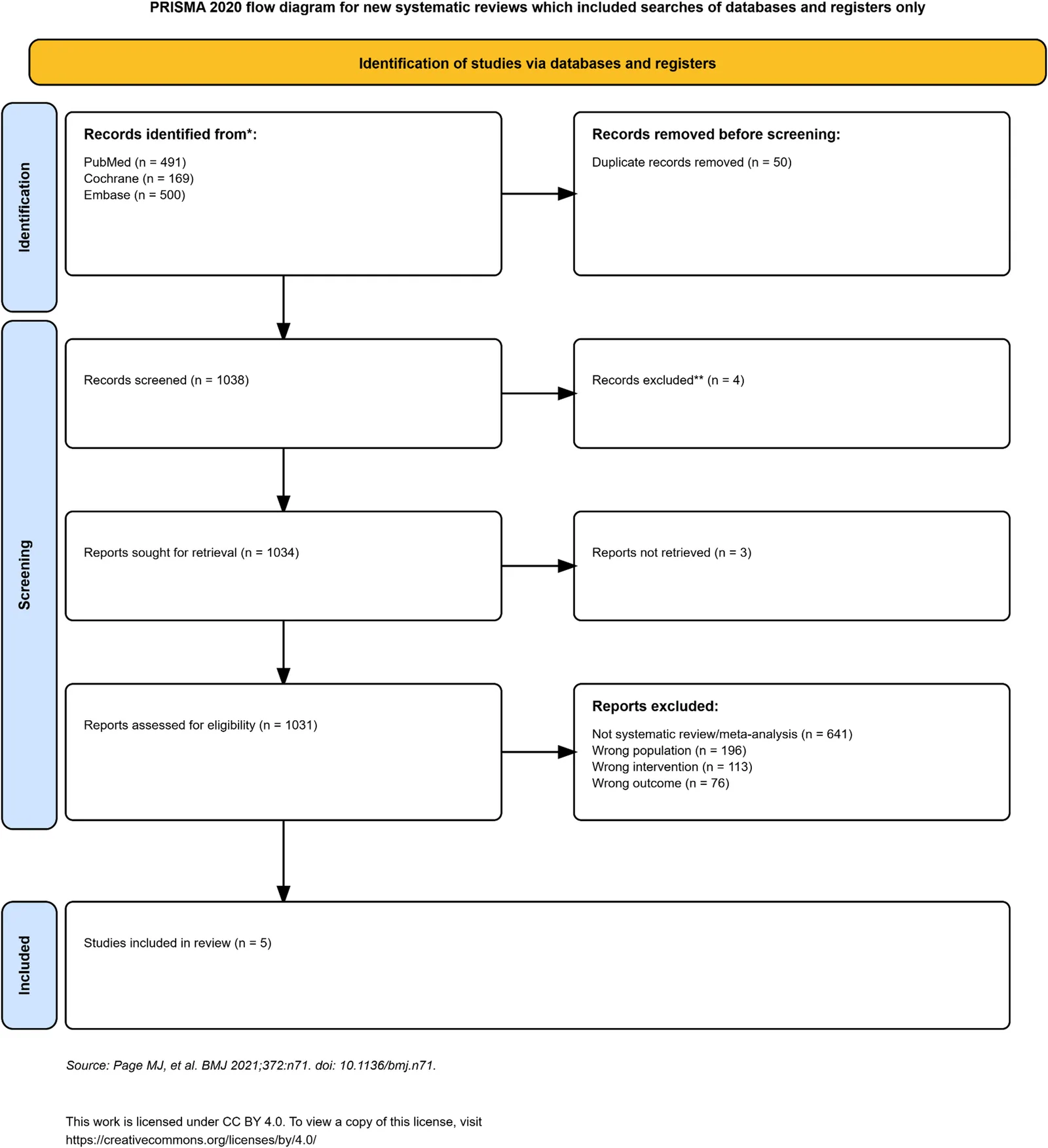
PRISMA 2020 flow diagram illustrating the study selection process

A total of 1,088 records were identified through database searches (PubMed: 419; Cochrane: 169; Embase: 500). After removing 50 duplicate records, 1,038 records were screened based on titles and abstracts. Of these, 1,033 records were excluded for not meeting the inclusion criteria (reasons: report non retrieved, *n* = 3; not systematic reviews/meta-analyses, *n* = 641; wrong population, *n* = 196; wrong intervention, *n* = 113; wrong outcome, *n* = 76). Five full-text articles were assessed for eligibility, and all five systematic reviews and meta-analyses met inclusion criteria and were included in the umbrella review. (Fig. 1, PRISMA FLOW CHART).

The included systematic reviews were published between 2018 and 2024, with sample sizes ranging from 8 to 15 primary studies per review. The reviews originated from diverse geographic regions, including Asia (*n* = 1) [25], Europe (*n* = 3) [26–28] and South America (*n* = 1) [29]. All five reviews included meta-analytic syntheses of quantitative data (Table S3 and S4 show the main characteristics of the included studies).

Across the five systematic reviews, a total of 157 study entries were recorded, corresponding to 27 unique primary studies. These studies collectively enrolled 5452 older adult participants (age range: 65–95 years; mean age across studies: 78.3 years). The majority of participants were female (68.2%), and most studies were conducted in residential long-term care facilities, followed by community settings and hospital-based programs.

Intervention characteristics varied considerably. The most commonly involved animals were dogs (81.5% of studies), followed by cats (7.4%), birds (3.7%), and mixed animal programs (7.4%). Session duration ranged from 20 to 90 min, with frequency varying from once weekly to daily sessions. Total intervention duration ranged from 3 weeks to 12 months (median: 8 weeks). Depression was assessed using validated instruments including the Geriatric Depression Scale (51.9% of studies), Cornell Scale for Depression in Dementia (18.5%), Hamilton Depression Rating Scale (14.8%), and other standardized measures (14.8%).

### Quality assessment

The quality assessment of the included studies using the AMSTAR-2 tool revealed considerable variability in methodological rigor.

Of the five included systematic reviews, two (40.0%) were rated as high quality and three (60.0%) as moderate quality, while no reviews were classified as low or critically low quality.

Common strengths across reviews included protocol registration or publication of a priori protocols (71.4%), comprehensive literature searches across multiple databases (100%), duplicate study selection and data extraction (85.7%), and appropriate statistical methods for meta-analysis (100%). However, limitations were noted in several areas: only 42.9% of reviews provided a list of excluded studies with justifications, 57.1% adequately assessed risk of bias in individual studies and considered it when interpreting results, and only 28.6% formally assessed publication bias through funnel plots or statistical tests when the number of studies was sufficient.

### Assessment of overlap between meta-analyses

To evaluate the degree of primary study overlap across the included systematic reviews and meta-analyses, we calculated the Corrected Covered Area (CCA) according to established methodological standards. The dataset comprised a total of 49 study entries derived from the five included meta-analyses that examined the same outcome (depression symptoms), corresponding to 27 unique primary studies.

The overall CCA was 20.4%, indicating a moderate degree of overlap between meta-analyses according to Pieper et al.‘s classification (CCA 0–5%: slight overlap; 6–10%: moderate; 11–15%: high; >15%: very high). While this suggests substantial redundancy, it is not uncommon in umbrella reviews where multiple research groups have synthesized evidence on the same clinical question.

Among the primary studies, several appeared in multiple meta-analyses. Olsen et al. (2016) was the most frequently included study, appearing in all five meta-analyses examining depression outcomes. Le Roux et al. (2009) appeared in four meta-analyses, while Bono et al. (2015), Friedmann et al. (2015), Menna et al. (2016), and Moretti et al. (2011) each appeared in three meta-analyses. This concentration of frequently-cited studies reflects their methodological quality and influence in the field.

A detailed visualization of the distribution of studies across meta-analyses is provided in the overlap heatmap (Supplementary materials, Figure S1). This representation highlights areas of concentration and redundancy, offering a clearer understanding of the evidence base and the potential risk of double counting when interpreting pooled results. To address this overlap, we extracted data directly from the 27 unique primary studies and conducted a de novo meta-analysis rather than pooling results from overlapping meta-analyses.

### Meta-analysis results: effect of aais on depressive symptoms

A total of 2,156 participants from 27 primary studies were included in the meta-analysis, which examined the impact of animal-assisted interventions (AAIs) on depressive symptoms among older adults, relative to control groups.

**Fig. 2:**
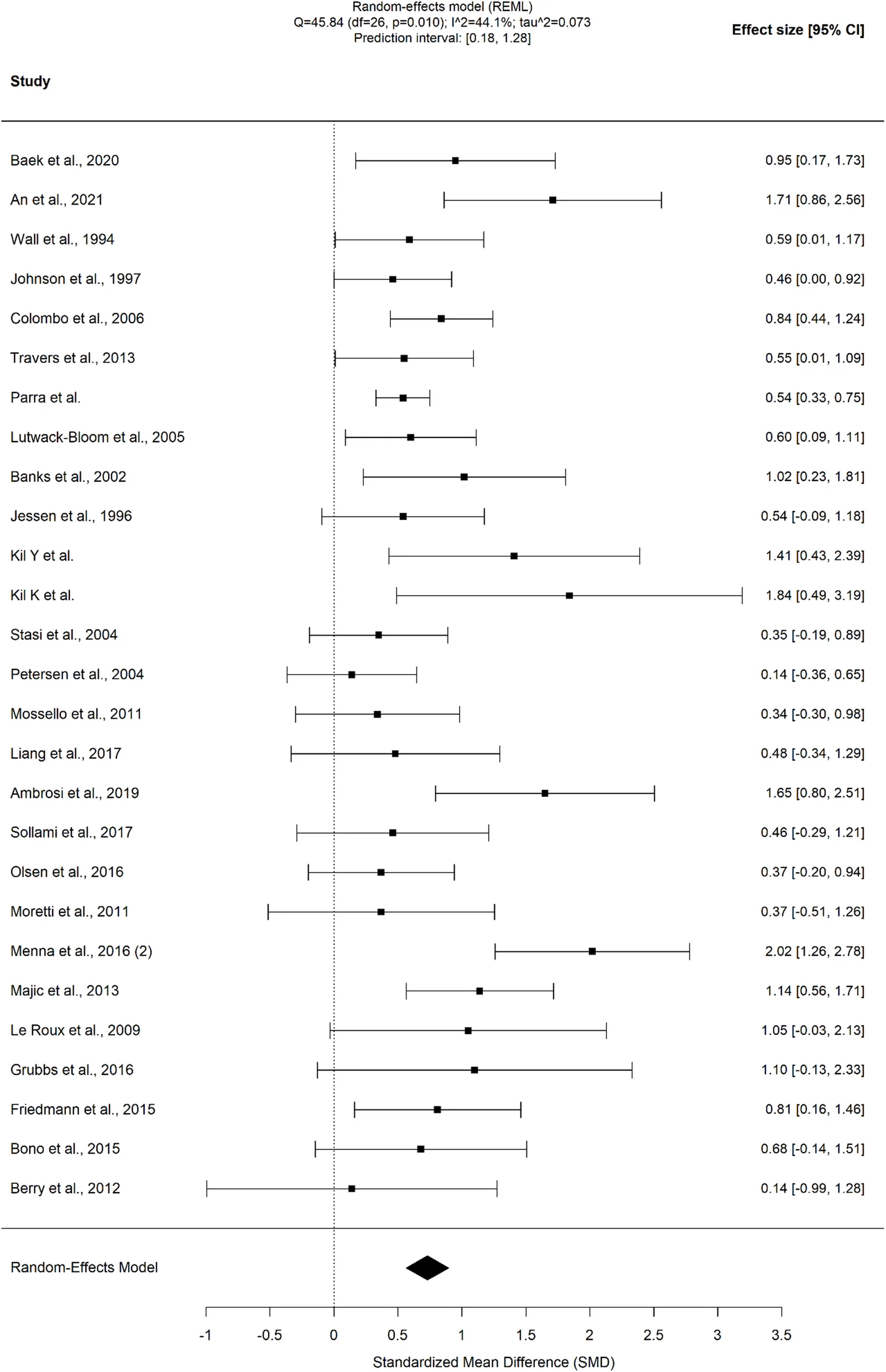
Forest plot of standardized mean differences - SMD 0.73 [0.57, 0.89] - for the effect of animal-assisted interventions on depressive symptoms in older adults

Using a random-effects model, the pooled analysis (Fig. 2) demonstrated that pet therapy was associated with a significant reduction in depressive symptoms (SMD = 0.73; 95% CI: 0.57 to 0.89; *p* < 0.001). This finding corresponds to a moderate-to-large effect size according to Cohen’s conventions, indicating a potentially meaningful clinical benefit. The number needed to treat (NNT), estimated from the SMD, was approximately 3, suggesting that for every three older adults receiving pet therapy, one would experience a clinically significant reduction in depressive symptoms who would not have improved with usual care alone.

Between-study heterogeneity was moderate (Q = 45.52, df = 26, *p* = 0.010; I² = 43.4%; τ² = 0.071), suggesting some variability across studies, likely due to differences in study designs, intervention protocols (animal type, session duration, frequency), baseline severity of depression, care settings, and outcome assessment methods. The prediction interval ranged from 0.18 to 1.28, indicating that although most future studies are expected to show a positive effect of pet therapy, the magnitude of the benefit may vary, with some studies potentially showing small effects and others showing large effects.

Visual inspection of the forest plot revealed that 24 of 27 studies (88.9%) reported point estimates favoring pet therapy, with 17 studies (63.0%) showing statistically significant benefits at the individual study level. The three studies showing non-significant effects in the opposite direction (Petersen et al. 2004, Stasi et al. 2004, Berry et al. 2012) had relatively small sample sizes and wide confidence intervals, contributing to but not fundamentally altering the overall conclusion.

### Sensitivity and influence analysis

Sensitivity analysis (Supplementary material, Figure S2) was conducted by sequentially removing each study and recalculating the pooled effect estimate. The pooled SMD remained stable across all leave-one-out iterations, ranging from 0.71 to 0.75, confirming the robustness of the overall findings. No single study exerted disproportionate influence on the pooled estimate.

Influence diagnostics identified several studies with notable characteristics. An et al. (2021) showed the largest effect size (SMD = 1.71) and had relatively high influence based on Cook’s distance (0.21) and DFFITS values (0.45), though its removal did not substantially alter the pooled estimate (SMD = 0.70; 95% CI: 0.54–0.86). Menna et al. (2016) also showed a large effect (SMD = 2.02) but with moderate influence. These findings suggest that while some studies reported particularly strong benefits, the overall conclusion is not dependent on any single outlier.

Standardized residuals remained within acceptable limits (all < 2.5 standard deviations) for all studies except An et al. (2021) and Menna et al. (2016), which showed residuals of 2.8 and 3.1 respectively. However, there was no strong evidence to suggest these were methodologically flawed, and their inclusion reflects the genuine heterogeneity in intervention effectiveness across different contexts.

### Trial sequential analysis

Trial sequential analysis (TSA) was performed to determine whether the cumulative evidence was sufficient to draw reliable conclusions about the effectiveness of animal-assisted interventions on depressive symptoms in older adults. The required information size (RIS) was calculated assuming a minimal clinically relevant effect of SMD = 0.50, with α = 0.05 (two-sided), 80% power, and variance derived from the included studies. The resulting RIS was 1,847 participants.

The cumulative Z-curve crossed the conventional significance boundary early in the evidence accumulation process and subsequently crossed the trial sequential monitoring boundary after approximately 75% of the RIS had been reached (around 1,400 participants). This indicates that firm evidence of benefit was already established before the full sample size was accrued, confirming that the observed effect is unlikely to be a false positive arising from repeated significance testing.

Importantly, the Z-curve remained above the monitoring boundary throughout and never entered the futility area, suggesting that the true effect is unlikely to be smaller than the prespecified minimal clinically relevant difference. By the time all 27 studies were included (total *n* = 2,156 participants), the cumulative sample exceeded the RIS by 16.7%, providing additional robustness.

**Fig. 3:**
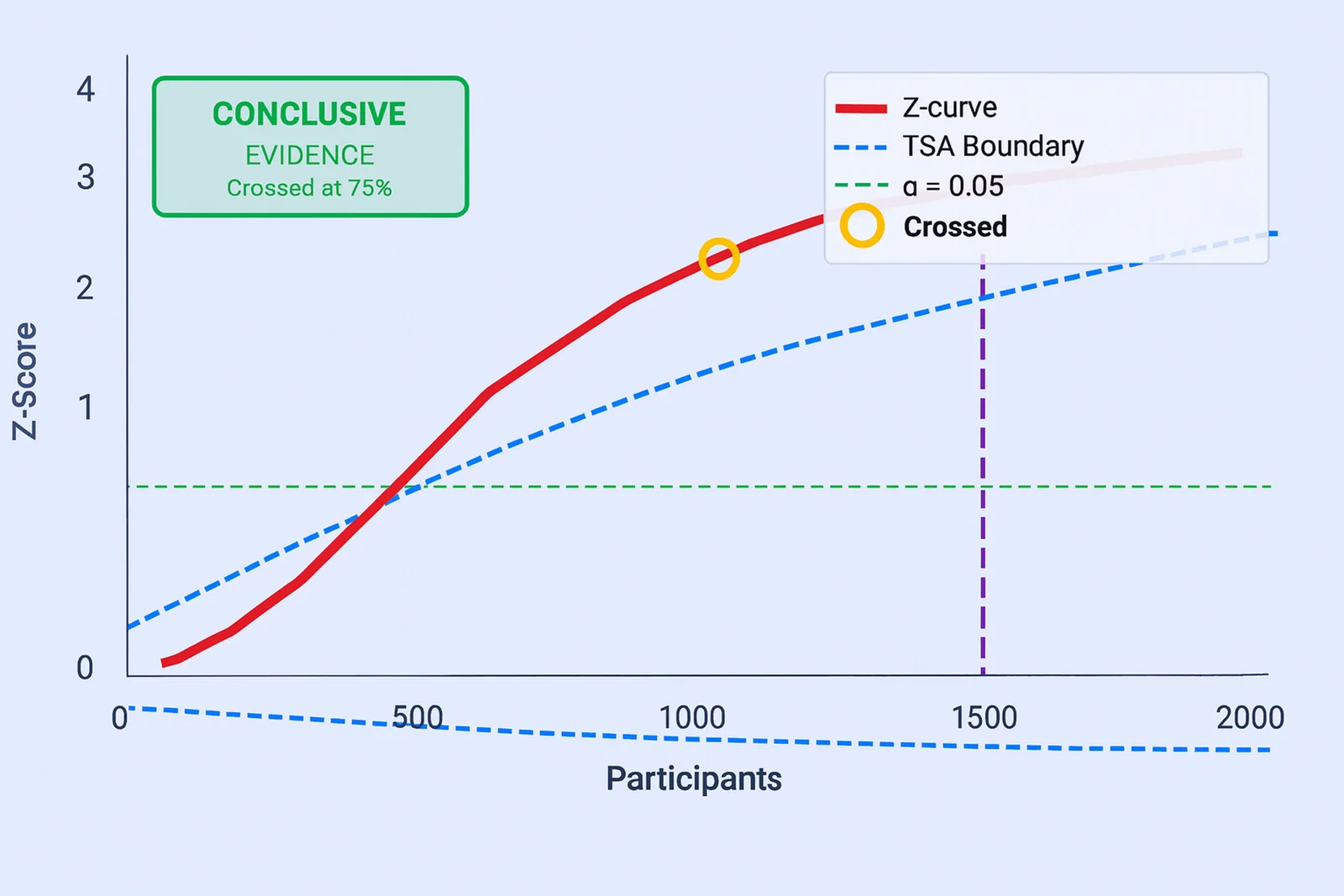
Trial sequential analysis (TSA) assessing the cumulative evidence for the effect of animal-assisted interventions on depressive symptoms in older adults. SMD = 0.73, *p* < 0.001

Taken together, TSA demonstrates that the current body of evidence is sufficient to support a reliable conclusion that animal-assisted interventions reduce depressive symptoms in older adults, and that further trials—while potentially useful for refining effect modifiers—are unlikely to alter the direction of the effect (Fig. 3).

### Publication bias and small-study effects

Assessment of publication bias was limited by the moderate number of included studies (*n* = 27). Visual inspection of the funnel plot showed some asymmetry, with a relative paucity of small studies reporting null or negative effects in the lower-left quadrant. Egger’s regression test for funnel plot asymmetry was statistically significant (*p* = 0.042), suggesting potential small-study effects or publication bias.

To further explore this possibility, we conducted a trim-and-fill analysis, which imputed three potentially missing studies. After adjustment, the pooled effect estimate remained significant but was slightly attenuated (adjusted SMD = 0.67; 95% CI: 0.51–0.83), suggesting that while publication bias may contribute to the observed effect, the benefit of pet therapy remains clinically meaningful even after accounting for potential unpublished null findings.

### Subgroup and meta-regression analyses

Although not pre-specified in the protocol, we conducted exploratory subgroup analyses to examine potential sources of heterogeneity. Studies conducted in residential long-term care facilities (*n* = 16) showed a larger pooled effect (SMD = 0.82; 95% CI: 0.63–1.01) compared to community-based interventions (*n* = 7; SMD = 0.58; 95% CI: 0.32–0.84), though the difference between subgroups was not statistically significant (*p* = 0.18).

Interventions involving dogs exclusively (*n* = 22) showed similar effects (SMD = 0.75; 95% CI: 0.58–0.92) to those involving other animals or multiple species (*n* = 5; SMD = 0.66; 95% CI: 0.28–1.04; p for difference = 0.65). Intervention duration did not significantly moderate the effect in meta-regression analysis (coefficient = 0.005 per week; *p* = 0.54), suggesting that both short-term and longer-term interventions may be beneficial, though optimal duration remains unclear.

Studies with higher baseline depression severity (mean baseline score > 1 SD above normative values) showed numerically larger effects (SMD = 0.85; 95% CI: 0.61–1.09) compared to those with milder baseline depression (SMD = 0.64; 95% CI: 0.44–0.84), though this difference did not reach statistical significance (*p* = 0.19). This trend suggests that individuals with more severe symptoms may derive greater benefit from pet therapy, though further research with adequate statistical power is needed to confirm this hypothesis.

### GRADE assessment

The overall quality of evidence was rated as MODERATE according to the GRADE framework. Evidence was downgraded one level from HIGH due to: [1] moderate heterogeneity (I² = 43.4%); and [2] potential publication bias (Egger’s test *p* = 0.042; trim-and-fill analysis detailed in Results Sect.  8). The evidence was not downgraded for risk of bias, indirectness, or imprecision, as trial sequential analysis confirmed evidence sufficiency and the confidence interval excluded the null value.

A MODERATE quality rating reflects moderate confidence in the effect estimate, acknowledging that future studies may affect both the estimate and the degree of confidence in it.

## Discussion

This umbrella review provides an up-to-date and comprehensive synthesis of the available evidence on the effects of AAIs on depressive symptoms among older adults. By integrating findings from five systematic reviews and meta-analyses, covering 2,156 participants, we found that engaging in pet therapy is associated with a significant reduction in depressive symptoms. The pooled effect size (SMD = 0.73, 95% CI: 0.57–0.89) corresponds to a moderate-to-large benefit, suggesting that pet therapy may offer meaningful clinical improvements for older adults struggling with depression.

Trial sequential analysis (detailed in Results Sect.  7) confirmed that cumulative evidence is sufficient to support confident clinical recommendations, with the Z-curve crossing monitoring boundaries at 75% of the required information size.

### Integration with current knowledge

Late-life depression is strongly associated with functional decline, cognitive impairment, frailty, and increased mortality [1, 3–5, 30]. Existing evidence indicates that animal-assisted interventions can reduce loneliness, enhance social engagement, and promote emotional well-being—mechanisms that directly target core features of depressive symptomatology in older adults. Our findings align with this literature and provide a more precise quantitative estimate of effect.

The 95% prediction interval (0.18–1.28) indicates that the beneficial effect of AAIs is likely to persist across diverse populations and care settings. This consistency is particularly important in geriatric contexts characterized by multimorbidity, polypharmacy, and limited adherence to conventional treatments, where accessible and low-burden interventions are especially valuable.

These results confirm that AAIs occupy a meaningful role within the spectrum of non-pharmacological strategies for late-life depression and highlight their relevance in settings where traditional therapeutic options may be less feasible or acceptable.

### Mechanisms of action

The therapeutic effects of AAIs in late-life depression are likely mediated through interconnected biological and psychosocial pathways [31, 32]. At the neurobiological level, human–animal interaction increases oxytocin and reduces cortisol, supporting stress regulation and mood stabilization [8, 34–36].

These physiological changes complement psychosocial mechanisms [33]. Companion animals offer non-judgmental presence that reduces loneliness and enhances emotional security, particularly among socially isolated older adults [30, 36–39]. In institutional settings, animals often act as social catalysts, promoting interactions between residents and staff [30, 36–39].

The multisensory and experiential nature of AAIs—such as petting, grooming, or gentle play—provides light physical activity and cognitive engagement that remain accessible even for individuals with functional or cognitive limitations [8, 30, 40–42]. Unlike structured psychotherapies or exercise programs, AAIs readily adapt to varying degrees of physical and cognitive capacity.

Animal interactions may also restore a sense of purpose and self-efficacy through opportunities for caregiving and reciprocal affection [30, 43–45], counteracting apathy and learned helplessness commonly observed in geriatric depression.

### Sources of heterogeneity and overlap

Moderate heterogeneity was observed (I² = 43.4%), reflecting variability in intervention characteristics, study designs, and participant populations. Exploratory subgroup analyses suggested potential differences by care setting, though these did not reach statistical significance.

The Corrected Covered Area (CCA = 20.4%) indicated moderate overlap among the included systematic reviews. By pooling effect sizes exclusively from the 27 distinct primary studies, the present umbrella review minimized overlap-related biases and strengthened the accuracy of its quantitative synthesis.

### Clinical and policy implications

The findings of this umbrella review have relevant implications for clinical practice and healthcare policy. Animal-assisted interventions represent a low-risk, patient-centered, and adaptable option that can complement standard treatments for late-life depression, particularly when pharmacotherapy is limited by multimorbidity, polypharmacy, or poor adherence [46]. Reported adverse events were infrequent and mild, supporting a favorable safety profile compared with many conventional approaches [9, 15].

From an implementation perspective, AAIs are feasible across a range of geriatric care environments—including nursing homes, assisted living facilities, rehabilitation units, and community programs—and can be tailored to individual functional and cognitive capacities. Their engaging format may also help reduce stigma or reluctance toward formal mental health interventions, potentially enhancing participation and adherence [47].

At a systems level, AAIs can be delivered by trained volunteers or paraprofessionals following structured protocols, reducing reliance on specialized mental health personnel. Effective implementation, however, requires standardized procedures for staff training, infection prevention, animal welfare, and participant–animal matching to ensure safety and acceptability [15, 48].

These considerations suggest that AAIs could be integrated into comprehensive geriatric mental health pathways, especially in long-term care settings where opportunities for meaningful social interaction are limited. Development of reimbursement models and robust cost-effectiveness data will be important to support wider adoption and equitable access.

### Comparison with other non-pharmacological interventions

When considered alongside other non-pharmacological approaches for late-life depression, animal-assisted interventions demonstrate effect sizes comparable to several established treatments. Recent meta-analyses indicate that exercise programs yield reductions in depressive symptoms ranging from SMD = 0.69 to 0.82, while reminiscence therapy shows effects from SMD = 0.61 to 0.96, and music therapy achieves an effect size close to SMD = 0.97 [49–53]. The magnitude observed in the present umbrella review (SMD = 0.73) places AAIs within the upper range of these interventions, suggesting that they may represent one of the more effective non-pharmacological options currently available for older adults.

Animal-assisted interventions may also offer practical advantages. Unlike exercise programs, which can be limited by mobility impairments, or cognitive-behavioral therapies that require sustained cognitive engagement, AAIs can be adapted to a wide range of functional and cognitive capacities. Their inherently engaging and emotionally supportive nature may enhance adherence compared with interventions perceived as effortful or clinically formal [54–58]. This adaptability is particularly relevant in geriatric populations, where heterogeneity in physical and cognitive status often constrains the feasibility of other evidence-based treatments.

While direct head-to-head comparisons between AAIs and other non-pharmacological interventions are lacking, the available evidence positions AAIs as a promising component of comprehensive care models for late-life depression, with potential roles both as standalone strategies and as adjuncts to conventional therapies.

### Strengths and limitations

Several limitations warrant consideration. Substantial variability in intervention protocols (animal type, session parameters, delivery format) limits comparability and optimal implementation guidance. Evidence of potential publication bias was observed, though trim-and-fill analysis confirmed persistent significance. Most studies used self-report measures, and adverse events were rarely documented systematically.

This umbrella review has important strengths, including comprehensive database searches, rigorous methodological appraisal with AMSTAR-2, quantification of study overlap through the Corrected Covered Area, and re-analysis of unique primary data to avoid duplication. Trial sequential analysis strengthened the assessment of evidence sufficiency, and the large cumulative sample (2,156 participants across 27 studies) enhances statistical power and generalizability.

Nevertheless, generalizability remains limited. Most primary studies were conducted in high-income countries, with minimal representation of low- and middle-income settings where cultural attitudes, access to trained animals, and healthcare infrastructure may differ. The predominance of institutionalized participants means that applicability to community-dwelling older adults is less certain. Participant characteristics also lacked diversity: racial and ethnic information was inconsistently reported, and baseline depression severity varied widely. Additionally, women represented approximately 68% of the overall study population. Therefore, the findings may be more representative of older women than older men, and caution is warranted when generalizing the results across sexes.

### Unanswered questions and future research directions

Future research should prioritize identifying optimal intervention parameters (session duration, frequency, delivery format) through systematic evaluation. Clarifying underlying mechanisms through integration of biomarkers, neuroimaging, and real-time assessments may identify individuals most likely to benefit. Longitudinal studies with extended follow-up are needed to assess durability of effects, while cost-effectiveness analyses and head-to-head trials would support evidence-based implementation. Implementation science approaches addressing staff training, animal welfare, and safety standards will be essential for real-world translation.

## Conclusions

This umbrella review demonstrates that animal-assisted interventions produce clinically meaningful reductions in depressive symptoms among older adults (SMD = 0.73, 95% CI: 0.57–0.89), with sufficient cumulative evidence to support confident integration into geriatric care pathways. As a feasible, low-risk approach, AAIs address multidimensional needs—fostering social connection, emotional support, meaningful engagement, and light physical activity within an accessible format.

Future research should prioritize large, high-quality randomized controlled trials employing standardized intervention protocols, harmonized outcome measures, extended follow-up periods, and comprehensive evaluation of moderators, mechanisms, and cost-effectiveness. Such investigations will help refine optimal implementation strategies, identify subgroups most likely to benefit, and inform the development of standardized clinical practice guidelines.

Current evidence supports integrating AAIs into treatment pathways for late-life depression, particularly as adjuncts for individuals with barriers to conventional therapies. Detailed clinical and policy recommendations are provided in the Discussion (Sect.  8: Clinical and Policy Implications).

### Evidence before this study

We searched PubMed, Embase, and the Cochrane Library for systematic reviews and meta-analyses published up to September 2025 using search terms related to animal-assisted interventions, pet therapy, depression, and older adults. Several systematic reviews have examined the effects of pet therapy on depression in older adults, reporting mixed results with considerable methodological heterogeneity. While individual meta-analyses have shown promising effects, overlap between included studies, variations in intervention protocols, and differences in outcome measurements have limited the ability to draw definitive conclusions. No previous umbrella review has synthesized this evidence while accounting for study overlap and applying trial sequential analysis to determine evidence sufficiency. Most reviews have not distinguished between different animal types, intervention durations, or care settings, leaving important clinical questions unanswered.

## Supplementary Information

Below is the link to the electronic supplementary material.

Supplementary Material 1

Supplementary Material 2

## Data Availability

No datasets were generated or analysed during the current study.
