# Supplementary material for: Animal-assisted interventions for depression in older adults: an umbrella review with meta-analysis and trial sequential analysis
Source: Aging Clin Exp Res. 2026 Jul 4;38(1):197. doi: 10.1007/s40520-026-03454-9 (PMC13612659; doi:10.1007/s40520-026-03454-9)
Supplement: Supplementary file 1 [file 40520_2026_3454_MOESM1_ESM.pdf]

Study	Overall	PICO	Protocol	Justificati	Compreh	Study	Data	List of	Descripti	Risk of	Sources	Appropri	Assessme	Investigat	Assessme	Conflict	Consider
Borgi et	**Moderat**	Partial/U	Yes	Yes	Yes	Yes	Yes	Yes	Yes	Yes	Yes	Yes	Partial/U	Yes	Yes	Yes	Yes
Orr et al.,	**Moderat**	Partial/U	Yes	Yes	Yes	Yes	Yes	Yes	Yes	Yes	Yes	Yes	Yes	Yes	Partial/U	Yes	Yes
Villarreal-	**High**	Yes	Yes	Yes	Yes	Yes	Yes	Yes	Yes	Yes	Yes	Yes	Yes	Yes	Yes	Yes	Yes
Ju Chang	**High**	Yes	Yes	Yes	Yes	Yes	Yes	Yes	Yes	Yes	Partial/U	Yes	Yes	Yes	Yes	Yes	Yes
Jain et al.,	**Moderat**	Partial/U	Yes	Partial/U	Yes	Yes	Yes	Yes	Yes	Yes	Yes	Yes	Yes	Yes	Yes	Yes	Yes

Supplementary Table S1. AMSTAR-2 methodological quality assessment of included systematic reviews. Detailed item-level Assessment of Multiple Systematic Reviews (AMSTAR-2) ratings for each of the five systematic reviews and meta-analyses included in the umbrella review. The table reports judgments across all 16 AMSTAR-2 domains, including protocol registration, adequacy of the literature search, justification for included study designs, duplicate study selection and data extraction, risk of bias assessment, appropriateness of meta-analytic methods, consideration of risk of bias in result interpretation, assessment of publication bias, and reporting of conflicts of interest. Overall confidence ratings (high, moderate, low) are derived according to AMSTAR-2 guidance based on the presence of critical and non-critical weaknesses.

Study	Risk of bias	Inconsistency	Indirectness	Imprecision	Publication bias	Overall certainty (GRADE)
Borgi et al., 2018	Not serious	Moderate	Not serious	Not serious	None	High ●●●●
Ju Chang et al., 2020	Serious	Moderate	Not serious	Serious	None	Moderate ●●●○
Jain et al., 2020	Serious	Moderate	Not serious	Not serious	None	Moderate ●●●○
Orr et al., 2023	Serious	Serious	Not serious	Serious	None	Low ●●○○
Villarreal-Zegarra et al., 2024	Serious	None	Not serious	Not serious	None	Moderate ●●●○

Supplementary Table S2. GRADE evidence profile for the effect of animal-assisted interventions on depressive symptoms. GRADE (Grading of Recommendations, Assessment, Development and Evaluation) summary of findings table evaluating the certainty of evidence for the effect of animal-assisted interventions on depressive symptoms in older adults. The table presents judgments across the five GRADE domains (risk of bias, inconsistency, indirectness, imprecision, and publication bias), along with pooled effect estimates (standardized mean difference with 95% confidence interval), number of participants and studies, and the resulting overall certainty of evidence rating. Downgrading decisions reflect observed heterogeneity and evidence of potential publication bias.

Villarreal-Zegarra et al.	Ju Chang et al.	**Author(s)**
2023	2020	**Year**
Peru	South Korea	**Country**
Systematic Review and Meta-analysis	Systematic Review and Meta-analysis	**Type of study**
Scopus, Web of Science, PubMed	Medline, Embase, Cochrane Library, Web of Science, SCOPUS, CINAHL, ProQuest	**Database**
Random-effects meta-analysis	Random-effects meta-analysis	**Analysis**
23	47	**Included studies (n)**
13	14	**Included studies (n) in meta-analysis**
1219	977	**Patients (n)**
65 or older	60 and older	**Age (y)**
Cochrane risk-of-bias tool for randomised trials (RoB2)	Critical appraisal tools of the Joanna Briggs Institute	**Quality assessment**
1) Effectiveness of animal-assisted therapy in reducing depression in older adults	Effect of animal-assisted therapy on depressive symptoms in elderly	**Main outcome (primary and secondary)**
AAT significantly reduced depressive symptoms compared to control	AAT significantly reduced depressive symptoms compared to control	**Main findings**
SMD = -0.72 (95% CI: -1.13, -0.31)	SMD = -1.31 (95% CI: -1.90, -0.72)	**Effect Size (95% CI)**
$p = 0.001$	$p < 0.001$	**Statistical Significance**

Jain et al.		
2020		
UK		
Systematic Review and Meta-analysis		
MEDLINE, EMBASE, PsycINFO, (Ovid), CINAHL, British Education Index, AMED, Social Policy and Practice, Web of Science, Social Care Online, SCOPUS, ERIC, Proquest, International Bibliography of the Social		
Random-effects meta-analysis		
43		
4	5	6
938		
65 and over		
Mixed methods appraisal tool (MMAT)		
2) Effect of dog-assisted intervention (versus treatment as usual) on activities of daily living or	1) Effect of dog-assisted intervention (versus treatment as usual) on depressive or loneliness	2) Effectiveness of PRI (pet robot intervention) in reducing depression in older
no overall effect of DAI on increasing activities of daily living or physical functioning	moderate significant effect of DAI on reducing depressive or loneliness symptoms	PRIs do not show a significant effect on reducing depressive symptoms
SMD = -0.11 (95% CI: -0.74, 0.52)	SMD = 0.66 (95% CI: 0.21, 1.11)	SMD = 0.01 (95% CI: -0.25, 0.27)
p = 0.737	p = 0.004	p = 0.93

Orr et al.				Borgi et al.
2023				2018
UK				Italy
Systematic Review and Meta-analysis				Meta-analysis
MEDLINE, Embase, APA PsycINFO, SPP (via Ovid), CINAHL Complete, AgeLine (EBSCOhost), CDSR, CENTRAL, DARE (Wiley Online, Cochrane Library), ASSIA (ProQuest), Web of Science Core				PubMed, Scopus
Random-effects meta-analysis				Random-effects meta-analysis
34				10
3	3	3	7	10
1986				332
65 and over				> 55
Wallace criteria and Cochrane Risk of Bias Tool				Downs and Black checklist, modified by Virués-Ortega and colleagues
4) Effect of animal-assisted therapy on	3) Effect of animal-assisted	2) Effect of animal-assisted therapy on anxiety	1) Effect of animal-assisted therapy on	Effect of dog-assisted interventions (DAI) on depressive symptoms in older adults
no evidence of effect from the dog visits on	no evidence that dog visits had an	small reduction in anxiety	no significant effect on reducing	DAI significantly reduce depressive symptoms
SMD = -0.06 (95% CI: -0.42, 0.30)	SMD = -0.31 (95% CI: -0.68, 0.07)	SMD = -0.36 (95% CI: -0.68, -0.04)	SMD = 0.34 (95% CI: -0.73, 0.04)	SMD = -1.002 (95% CI: 0.54, 1.45)
p = 0.75	p = 0.11	p = 0.03	p = 0.08	p < 0.001

Supplementary Table S3. Characteristics of included systematic reviews and meta-analyses. Summary of the main characteristics of the systematic reviews and meta-analyses included in the umbrella review, including first author and year of publication, geographic region, databases searched, number and type of primary studies included, total sample size, study designs, and primary outcomes assessed. This table provides an overview of the scope, methodological approaches, and evidence base of the included reviews.

Author(s)	Year	Type of study	Main outcome (primary and secondary)	Settings	Scale used	Animal	Time
Ju Chang et al.	2020	Systematic Review and Meta-analysis	Effect of animal-assisted therapy on depressive symptoms in elderly	home, nursing homes, day care centers, long-term care facilities, skilled rehabilitation units, community, medical care hospital, psychiatric hospital ward, Alzheimer's special care unit, residential care facilities	GDS, CSDD, BDI	Dog, robotic pet (PARO), horse (including horse riding simulation), toy/robotic or live cats, budgerigar, cricket, rabbit, kitten or puppy, guinea pig	most frequently once a week. The duration of intervention ranged from 1 day to 12 months; the most frequent time of one session was 30 minutes, with a range of 5–10 minutes to 180 minutes. Two studies were designed for the animals to stay continuously in the study setting
Villarreal-Zegarra et al.	2023	Systematic Review and Meta-analysis	1) Effectiveness of animal-assisted therapy in reducing depression in older adults	rehabilitation hospital, hospital setting, nursing homes, assisted living facilities, adult day care, day centre, care centre, residential age care facility, secure dementia units	BDI-II, CSDD, GDSSF-K, DMAS, GDS, GDS-SF, DASS-21, HAMD17	Dog, bird, cat, PRI (PARO)	The interventions varied from 10 to 90 min, during a time-lapse of 6 weeks to 8 months, and frequency was from one to three times per week.

Jain et al.	
2020	
Systematic Review and Meta-analysis	
1) Effect of dog-assisted intervention (versus treatment as usual) on depressive or loneliness symptoms among older adults	2) Effectiveness of PRI (pet robot intervention) in reducing depression in older adults
residential long-term care facilities	
GDS, UCLA-LS, BDI, CSDD, CMAI, MMSE, SAFE, BI, SF-36-Sub	
Dog	
most frequently once a week, for an intervention period of between 1 and 52 weeks. The duration of each visit ranged from 3 min to 3 hr	

Orr et al.	Borgi et al.	
2023	2018	
Systematic Review and Meta-analysis	Meta-analysis	
1) Effect of animal-assisted therapy on depression	Effect of dog-assisted intervention on depressive symptoms in older adults	2) Effect of dog-assisted intervention (versus treatment as usual) on activities of daily living or physical functioning among older adults
skilled nursing facilities, long-term care facilities, nursing homes, aged care facilities, long-term assisted living facilities	nursing homes, hospitals, assisted-living residences, retirement communities for older adults	
BSI, CSDD, MAACL-R, BDI, GDS, MS-E, BAI, CMAI, BARS, QOL-AD, QUALID, NPI, MOSES, LEIPAD-SV, PGC Morale	GDS, CSDD, DMAS, BDI	
Dog, canary, rabbit, kitten, robotic seal	Dog	
from 5-10 min/week to 2 X 90 min/week over a range of 3 to 12 weeks	mostly twice per week. Duration of the sessions ranged from 30 to 90 min and the length of the interventions ranged from 6 weeks to 8 months	

			2) Effect of animal-assisted therapy on anxiety					
			3) Effect of animal-assisted therapy on agitation					
			4) Effect of animal-assisted therapy on quality of life					

Supplementary Table S4. Characteristics of primary studies included in the de novo meta-analysis. Detailed description of the 27 unique primary studies included in the re-analysis, reporting study design, country, sample size, participant characteristics (age and sex distribution), care setting, type of animal-assisted intervention (animal species, session duration, frequency, and total intervention length), comparator conditions, depression assessment tools, and main outcome data used for quantitative synthesis. This table underpins the pooled effect estimates by documenting the individual study-level inputs.
