# Supplementary material for: Animal-assisted interventions for depression in older adults: an umbrella review with meta-analysis and trial sequential analysis
Source: Aging Clin Exp Res. 2026 Jul 4;38(1):197. doi: 10.1007/s40520-026-03454-9 (PMC13612659; doi:10.1007/s40520-026-03454-9)
Supplement: Supplementary file 2 [file 40520_2026_3454_MOESM2_ESM.docx]

**Supplementary materials**

### **Figure S1. Overlap heatmap of primary studies across included meta-analyses.** Heatmap illustrating the distribution and overlap of primary studies included in the five systematic reviews and meta-analyses retained in the umbrella review. Rows represent individual primary studies, while columns correspond to the included meta-analyses. Filled cells indicate inclusion of a given primary study within a specific meta-analysis. This visualization highlights areas of redundancy and concentration of evidence and supports the calculation of the Corrected Covered Area (CCA).

### **Figure S2. Influence and diagnostic plots for the random-effects meta-analysis.** Diagnostic plots assessing the influence of individual studies on the pooled effect estimate, including standardized residuals, DFFITS values, Cook’s distance, covariance ratios, leave-one-out tau² estimates, hat values, and study weights. These analyses were performed to identify influential or outlying studies and to evaluate the robustness of the pooled standardized mean difference.
